# *Schistosome* egg-derived extracellular vesicles deliver Sja-miR-71a inhibits host macrophage and neutrophil extracellular traps via targeting *Sema4D*

**DOI:** 10.1186/s12964-023-01395-8

**Published:** 2023-12-21

**Authors:** Yao Liao, Zifeng Zhu, Yuheng Liu, Ji Wu, Dinghao Li, Zhen Li, Junhao Xu, Ruibing Yang, Lifu Wang

**Affiliations:** 1 Guangzhou key laboratory for clinical rapid diagnosis and early warning of infectious diseases, KingMed School of Laboratory Medicine, Guangzhou Medical University, Guangzhou, 511436 Guangdong China; 2 Guangzhou KingMed Diagnostic Laboratory Ltd, Guangzhou, 510320 China; 3 Department of Parasitology of Zhongshan School of Medicine, Sun Yat-sen University, Guangzhou, 510080 China

**Keywords:** *Schistosoma japonicum* eggs, Extracellular vesicles, Sja-miR-71a, Extracellular traps, *Sema4D*

## Abstract

**Background:**

Macrophages and neutrophils are rapidly recruited around *Schistosome* eggs to form granulomas. Extracellular traps (ETs) of macrophages and neutrophils are part of the pathogen clearance armamentarium of leukocytes. *Schistosome* eggs possess the ability to resist attack by the host’s immune cells and survive by employing various immune evasion mechanisms, including the release of extracellular vesicles (EVs). However, the specific mechanisms by which *Schistosome* egg-derived EVs (E-EVs) evade the immune response and resist attack from macrophage and neutrophil ETs remain poorly understood. In this study, we aimed to investigate the association between E-EVs and macrophage/neutrophil ETs.

**Methods:**

EVs were isolated from the culture supernatant of *S. japonicum* eggs and treated macrophages and neutrophils with E-EVs and Sja-miR-71a. The formation of ETs was then observed. Additionally, we infected mice with *S. japonicum*, administered HBAAV2/9-Sja-miR-71a, and the formation of macrophage ETs (METs) and neutrophil ETs (NETs) in the livers was measured. Sema4D-knockout mice, RNA sequencing, and trans-well assay were used to clarify Sja-miR-71a in E-EVs inhibits METs and NETs formation via the *Sema4D*/ PPAR-γ/ IL-10 axis.

**Results:**

Our findings revealed that E-EVs were internalized by macrophages and neutrophils, leading to the inhibition of METs and NETs formation. The highly expressed Sja-miR-71a in E-EVs targeted Sema4D, resulting in the up-regulation of IL-10 and subsequent inhibition of METs and NETs formation. *Sema4D* knockout up-regulated IL-10 expression and inhibited the formation of METs and NETs. Furthermore, we further demonstrated that Sja-miR-71a inhibits METs and NETs formation via the *Sema4D*/ PPAR-γ/ IL-10 axis.

**Conclusions:**

In summary, our findings provide new insights into the immune evasion abilities of *Schistosome* eggs by demonstrating their ability to inhibit the formation of METs and NETs through the secretion of EVs. This study enhances our understanding of the host-pathogen interaction and may have implications for the development of novel therapeutic approaches.

Video Abstract

**Supplementary Information:**

The online version contains supplementary material available at 10.1186/s12964-023-01395-8.

## Background

Schistosomiasis is a neglected tropical disease that infects approximately 250 million people and causes 1.4-3.3 million disability-adjusted life years (DALYs) [1, 2]. Current treatment options for schistosomiasis rely on praziquantel, as no effective vaccines have been developed. Schistosome parasites employ various immunoregulatory mechanisms to counteract the host’s immune system and ensure their own survival [3]. *Schistosome* eggs are trapped in tissues and surrounded by granulomas composed of various cell types to prevent tissue damage. *Schistosome* eggs need to resist attack by host immune cells and maintain the possibility of translocation in order to survive and be released from the host’s body. *Schistosome* eggs release several factors, such as *S. mansoni* chemokine binding protein (smCKBP) and Omega-1, which influence immune cell recruitment and granuloma size [4, 5]. However, the precise mechanisms by which *Schistosome* eggs regulate host immune cells remain poorly understood.

Macrophages play an important role in the response to parasitic infection and are the primary way for the human body fights against worms [6]. Most macrophages found in the inflammatory sites caused by schistosomiasis are derived from blood monocytes [7]. Macrophage-dense epithelioid granulomas rapidly form around mature eggs [8]. Neutrophils, on the other hand, play a critical role in eliminating invading pathogens [9]. Following *Schistosome* cercariae infection, neutrophils are recruited to the infection site within 3 hours [10]. Neutrophils adhere to the *Schistosoma* surface in the presence of complement proteins and antibodies, thereby impairing its motility and development [11]. As the *Schistosome* eggs mature, they release antigens that stimulate the surrounding macrophages, neutrophils, and other immune cells to form granulomas [8]. These granulomas protect the host by sequestering toxic egg antigens and accelerating their death [12, 13]. However, for the parasite, the eggs ultimately need to break out of the granuloma to be released from the host and complete their life cycle.

Following strong activation signals, neutrophils and macrophages release DNA fibers and granular proteins into the extracellular space, forming extracellular traps (ETs) [14]. Initially recognized for their bactericidal and antifungal properties, ETs have also been implicated in parasite clearance. Macrophage ETs (METs) act as early effectors against the abortive parasite *Neospora caninum* [15]*.* Our previous study found that host liver-derived extracellular vesicles (EVs) deliver miR-142a-3p, which induces neutrophil ETs (NETs) to block the development of *Schistosoma japonicum* (*S. japonicum*) by targeting WASL [16]. Neutrophils are rapidly recruited and deploy NETs around skin-penetrating hookworm larvae; however, the hookworm can secrete a deoxyribonuclease that degrades NETs [17].

EVs secreted from *Schistosoma* are important pathways for *Schistosoma*-host communication. EVs derived from adult *S. japonicum* mediate the M1-type immune-activity of macrophages [18], and miRNA-carrying EVs derived from *S. mansoni* worms modulate host T helper cell differentiation [19]. Moreover, EVs derived from *Schistosome* eggs have been found to suppress liver fibrosis through the transport of Sja-miR-71a [13]. Based on these findings, we hypothesize that while macrophages and neutrophils form granulomas to encapsulate *Schistosome* eggs, the eggs themselves secrete EVs to hinder the function of these immune cells. In this study, we investigate the inhibitory effects of EVs secreted by *S. japonicum* eggs on the formation of METs and NETs. We further explore the role of the specific miRNA, Sja-miR-71a, carried by these EVs in modulating METs and NETs by targeting Sema4D.

## Methods

### Animal experiments

Male C57 BL/6 J mice (6 weeks old) were purchased from Guangdong Medical Laboratory Animal Center (Guanghzou, China). *Sema4D* gene KO mice (male, 6 weeks old, C57BL/6 J) were purchased from Cyagen Biology (Suzhou, China). Mice were infected via percutaneous exposure to *S. japonicum* cercariae (30 cercaria/per mouse) that were shed from *Oncomelania hupensis*. All animal experiments were approved by the Guangzhou Medical University Committee for Animal Research and conformed to the Guidelines for the Care and Use of Laboratory Animals of the National Institute of Health in China.

### EVs purification and identification

EVs were harvested as described in our previous study [13]. Briefly, *S. japonicum* eggs were collected from *S. japonicum* infected mice 45 days post-infection. The eggs were maintained in RPMI-1640 culture medium (Gibco, Germany). The culture supernatant was centrifuged at 700 g for 30 min at 4 °C (15 ml polypropylene tube, swinging bucket rotor, model A-4-44, 5804R Refrigerated Centrifuge, Eppendorf, Germany), and the resulting supernatant was centrifuged at 3500 g for 30 min at 4 °C (15 ml polypropylene tube, swinging bucket rotor, model A-4-44, 5804R Refrigerated Centrifuge, Eppendorf, Germany). That supernatant was centrifuged at 20,000 g for 60 min at 4 °C (Fixed angle rotor, angle is 45 degrees, model #3331, D-37520 Refrigerated Centrifuge, Thermo Electron Corporation, USA), and the resulting supernatant from that was centrifuged at 120,000 g for 90 min at 4 °C in an Optima L-100xp tabletop ultracentrifuge (Swinging bucket rotor, model SW40 Ti, Optima L-100xp, Beckman Coulter, USA). The resultant pellet (EVs) was diluted with phosphate-buffered saline (PBS). EVs were analyzed using negative-staining transmission electron microscopy (TEM). EVs were loaded on a copper grid and negatively stained with 3% (w/v) aqueous phosphotungstic acid for 1 min. The grid was examined using an FEI Tecnai G2 Sprit Twin TEM (FEI, USA). EV particles were also analyzed using nanoparticle tracking analysis (NTA) (NanoSight NS300, Malvern Instruments, United Kingdom).

### Macrophage and neutrophil isolation

The femurs and tibias of C57 BL/6 J mice were removed, placed in 75% ethanol (5 min) and then washed using Dulbecco’s modified Eagle’s medium (DMEM, Gibco, Germany). Cells within the bone marrow were prepared as a single-cell suspension. For macrophage isolation, cells within the bone marrow were cultured in DMEM with 10% (v/v) fetal bovine serum and recombinant murine macrophage colony-stimulating factor (20 ng/mL, Novoprotein, China). On the seventh day, bone marrow-derived macrophages (BMDMs) were harvested. For neutrophil isolation, cells were isolated from the single-cell bone marrow suspension using Percoll density gradient centrifugation and separated via positive selection for CD11b^+^Ly6G^+^ cells (Anti-CD11b antibody, BioLegend, USA, 101205; Anti- Ly6G antibody, Tonbo Biosciences, 20-5931) on flow cytometry (BD Influx, USA).

### EV uptake experiment

EVs were labelled with PKH26 (Sigma-Aldrich, USA) for 5 min at room temperature, and staining was terminated by adding an equal volume of 1% BSA. The liquid was then centrifuged at 120,000 g for 90 min at 4C in an Optima L-100xp ultracentrifuge (swinging-bucket rotor, model SW60 Ti, Optima L-100xp, Beckman Coulter) to remove unlabelled PKH26. The PKH26-labeled EVs were resuspended with PBS. Macrophages and neutrophils were incubated with the PKH26-labeled EVs for 1 h and then analyzed using confocal microscopy to evaluate EV internalization. Actin was labelled with Alexa Fluor phalloidin-FITC (CST), and DAPI was used to detect nuclei.

### Cell culture and treatment

Neutrophils and macrophages were cultured in a humidified, 5% CO2 incubator at 37 °C. They were then treated with *Schistosome* egg-derived EVs (E-EVs, 10 μg/ml) for 24 h, a Sja-miR-71a mimic (50 nM, Ribobio China) for 24 h, recombinant Sema4D protein (10 μg/ml; Abclonal, USA) for 24 h, IL-10 antibodies (15 μg/ml; Proteintech, China) for 24 h, and a PPAR-γ agonist (30μΜ; MedchemExpress, USA) and antagonist (20 μM; MedchemExpress, USA) for 24 h. phorbol-12-myristate-13-acetate (PMA) (500 nM; ETs-inducer; Sigma-Aldrich, USA) Reagent was used to stimulate neutrophils and macrophages 4 h before harvesting.

### Recombinant adeno-associated virus (rAAV) vectors and transduction

As described in our previous study [13], the rAAV HBAAV2/9-Sja-miR-71a was purchased from Hanbio Biotechnology Co., Ltd. Mice were randomized into groups and inoculated with rAAV (1.5 × 10 [11] v.g./mouse) via tail vein injection 10 days after infection with *S. japonicum*. The green fluorescent protein (GFP) expression associated with HBAAV2/9-Sja-miR-71a in the mouse liver was observed using an in vivo imaging system (PerKinElmer, USA) and fluorescence microscopy.

### Immunofluorescence analysis

Cells were fixed in 4% paraformaldehyde and permeabilized with 0.2% Triton X-100 for 10 min. After being blocked with 2% bovine serum albumin (BSA) for 30 min at room temperature, the cells were incubated with anti-H3Cit (1:400; CST, USA, 14269S), anti-MPO (1:100; Abcam, UK, ab208670), or anti-F4/80 (1:400; CST, USA, 30325) antibodies overnight at 4 °C. Liver tissues were fixed in 4% paraformaldehyde and embedded in paraffin. The paraffin sections were subsequently prepared and deparaffinized by baking, then dehydrated using xylene and ethanol. After blocking with 1% BSA for 60 minutes, the sections were incubated with anti-H3Cit (1:400; CST, USA), anti-MPO (1:100; Abcam, UK), or anti-F4/80 (1:400; CST, USA) antibodies overnight at 4 °C. The cells and liver sections were then incubated with the indicated Alexa Fluor-conjugated secondary antibodies. DAPI was used to detect nuclei. Between all steps, samples were washed three times with PBS for 5 min each. The sections were visualized using a LSM 800 laser scanning confocal microscope (Zeiss, Germany).

### Scanning electron microscopy

Macrophages and neutrophils were cultured on coverslips. Cells were fixed in 2.5% glutaraldehyde overnight and then washed with PBS before dehydration using an ethanol gradient. The ethanol was then exchanged for acetone and isoamyl acetate. Coverslips were critical point-dried and coated with gold using an ion coater (E102, Hitachi) and observed using an FEI Quanta 200 scanning electron microscope.

### RNA extraction and quantitative reverse-transcription PCR (qRT-PCR)

mRNA expression was quantified using qRT-PCR. In brief, RNA was extracted from cells and liver tissue using the TRIzol reagent (Invitrogen, USA) according to the manufacturer’s instructions. The extracted RNA was quantified using a NanoDrop ND-2000 spectrophotometer (Thermo Scientific, USA). Complementary DNA (cDNA) was synthesized from 1.0 μg of total RNA using the PrimeScript™ RT Master Mix (Perfect Real Time) (Takara, Japan) according to the manufacturer’s protocol. IL-10 and PPAR-γ expression was analyzed using TB Green® *Premix Ex Taq*™ II (Tli RNaseH Plus) (Takara, Japan). The primers used for qRT-PCR are listed in Supplementary Table S1. GAPDH was used as an internal control, and fold change was calculated using the 2^-ΔΔCT^ method.

### Western blotting

Cells and liver tissues were homogenized using RIPA lysis buffer in the presence of freshly added protease and phosphatase inhibitors (Thermo Fisher Scientific, USA). Lysates were then quantified and subjected to 10% sodium dodecyl-polyacrylamide gel electrophoresis. The resolved proteins were transferred to a polyvinylidene fluoride blotting membrane (GE Healthcare Life Sciences, UK). The membranes were blocked using 5% skim milk and then incubated with IL-10 (1:500; Proteintech, China) and PPAR-γ (1:1000; Proteintech, China, 60,269-1-Ig) antibodies. GAPDH (1:5000; Proteintech, China, 60,004-1-Ig) and β-actin (1:5000; Proteintech, China, 66,009-1-1 g) were used as internal standards. The membranes were visualized using an ECL western blot detection system (Amersham, USA).

### RNA sequencing and analysis

Total RNA was isolated from the liver tissues of wild-type and *Sema4D* knockout mice infected with *S. japonicum,* and then subjected to quantitative and qualitative analyses. A total of 3 μg of RNA from each sample was used to prepare the RNA library. Following cluster generation, the library samples were sequenced on an Illumina Hiseq 2500 platform (Illumina, USA). The sequencing data were subjected to q quality control analysis, read mapping to the reference genome, transcriptome assembly, coding potential analysis, conservative analysis, target gene prediction, gene expression level quantification, differential expression analysis, and Gene Ontology (GO) and Kyoto Encyclopedia of Genes and Genomes (KEGG) enrichment analysis.

### Quantification and statistical analysis

Results are expressed as mean ± SD. Differences between two groups were compared using unpaired two-sample *t* tests. Multiple comparisons between more than two groups were analyzed with one-way ANOVA. *P* < 0.05 was considered statistically significant. Immunofluorescence and scanning electron microscopy data for METs and NETs were quantified using Image J software (NIH, Bethesda, MD, USA). Statistical analyses were performed using GraphPad Prism 8.0 (LaJolla, CA, USA). The investigators were not blinded to allocation during experiments and outcome assessment. No exclusion criteria were applied to exclude samples from analysis.

## Results

### E-EVs were internalized by macrophages and neutrophils and inhibited METs and NETs formation

**Fig. 1:**
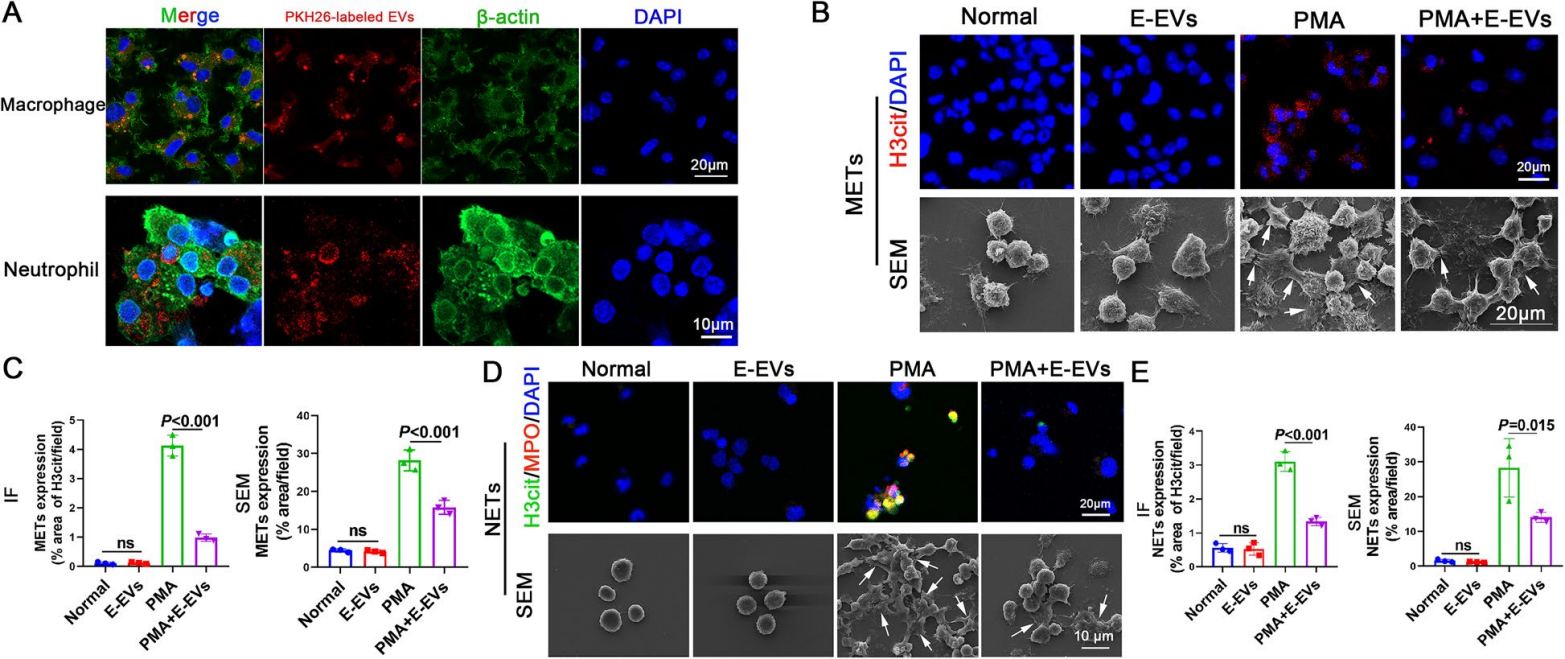
*S. japonicum* egg-derived extracellular vesicles (E-EVs) were internalized by macrophages and neutrophils and inhibited METs and NETs formation. **A** Macrophages and neutrophils were incubated with PKH26-labeled E-EVs, and E-EVs internalization was examined using laser scanning confocal microscopy. **B-E** Macrophages and neutrophils were treated with E-EVs (10 μg/mL, 24 h), PMA (500 nM, 5 h), or PMA (500 nM, 5 h) + E-EVs (10 μg/mL, 24 h). Macrophage extracellular traps (METs) and neutrophil extracellular traps (NETs) were observed using immunofluorescence (IF) and scanning electron microscopy (SEM) (**B** and **D**) and were quantified (**C** and **E**). METs were detected on IF based on H3cit. NETs were detected based on H3cit and MPO co-localization, Arrows: METs or NETs. IF was quantified based on area the of H3cit, and SEM was quantified based on the area of METs and NETs. e, g, representative of *n* = 3 independent experiments

E-EVs were isolated from the culture supernatant of *S. japonicum* eggs and analyzed using TEM. TEM revealed that EVs had diameters ranging from 50 to 150 nm and a characteristic cup-shaped morphology (Fig. S1A, arrows). The size distribution profile of the EVs was examined using NTA, which revealed a peak size of 129 nm (Fig. S1B). Remarkably, we found that E-EVs could be internalized by macrophages and neutrophils (Fig. 1A). Macrophages and neutrophils were treated with E-EVs, PMA (ETs inducers), or PMA + E-EVs, immunofluorescence (IF) and scanning electron microscopy (SEM) revealed that E*-*EVs inhibited the PMA-induced formation of METs and NETs (Fig. 1B-E). These data suggest that *S. japonicum* eggs secrete EVs to inhibit METs and NETs formation.

### Sja-miR-71a in E-EVs inhibits METs and NETs formation

**Fig. 2:**
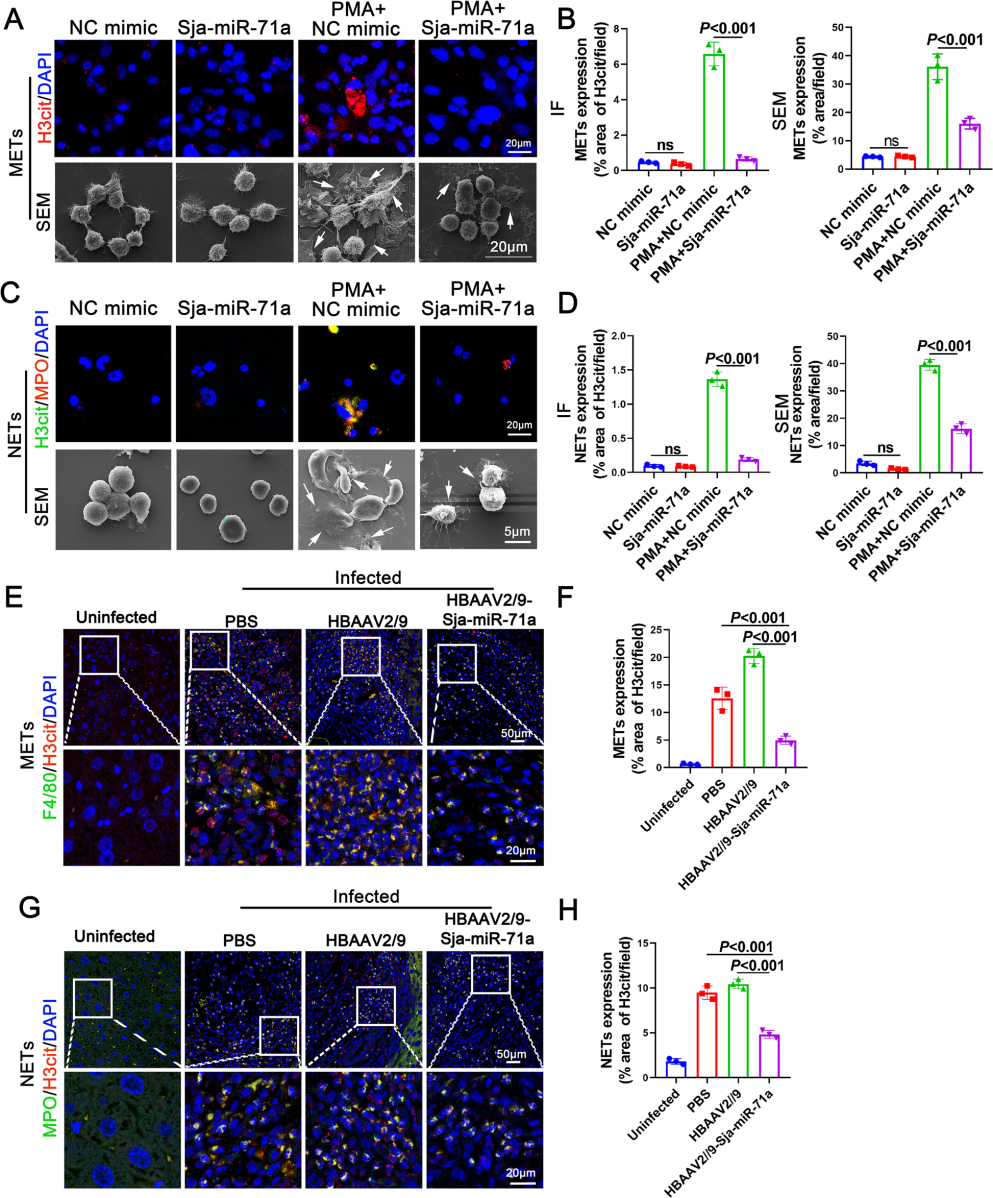
Sja-miR-71a in E-EVs inhibits METs and NETs formation. **A-D** Macrophages and neutrophils were treated with normal control (NC) mimic (50 nM, 24 h), Sja-miR-71a (50 nM, 24 h), PMA (500 nM, 5 h) + NC mimic (50 nM, 24 h), or PMA (500 nM, 5 h) + Sja-miR-71a (50 nM, 24 h). METs and NETs were observed using IF and SEM (**A** and **C**), and quantified (**B** and **D**). (**E-H**) METs (**E**) and NETs (**G**) in liver sections were detected and quantified (**F** and **H**). Arrows: METs or NETs. **B**, **D**, **F**, **H** representative of *n* = 3 independent experiments

Our previous studies have shown that Sja-miR-71a was most highly expressed in E-EVs miRNAs [13]. We sought to determine if E-EVs inhibition of METs and NETs formation was related to Sja-miR-71a. Interestingly, we found that E-EVs can deliver Sja-miR-71a to macrophages and neutrophils (Fig. S2A, B). Furthermore, Sja-miR-71a significantly suppressed the formation of METs induced by PMA (Fig. 2A, B) and also inhibited the formation of NETs (Fig. 2C, D). To further confirm the involvement of Sja-miR-71a delivered by E-EVs in METs and NETs formation in vivo, we constructed a HBAAV2/9-Sja-miR-71a recombinant adeno-associated virus (rAAV). Subsequently, mice infected with *S. japonicum* were administered HBAAV2/9-Sja-miR-71a (Fig. S3), and METs and NETs formation in the livers of mice was evaluated. Notably, METs were observed in *S. japonicum* granulomas (Fig. 2E, F), whereas a significant decrease in METs formation was observed in mice treated with HBAAV2/9-Sja-miR-71a (Fig. 2E, F). Similarly, NETs were observed in *S. japonicum* granulomas (Fig. 2G, H), and their formation was significantly decreased after treatment with HBAAV2/9-Sja-miR-71a (Fig. 2G, H). These results suggest that E-EVs deliver Sja-miR-71a to inhibit METs and NETs formation.

### E-EVs and Sja-miR-71a up-regulate IL-10

**Fig. 3:**
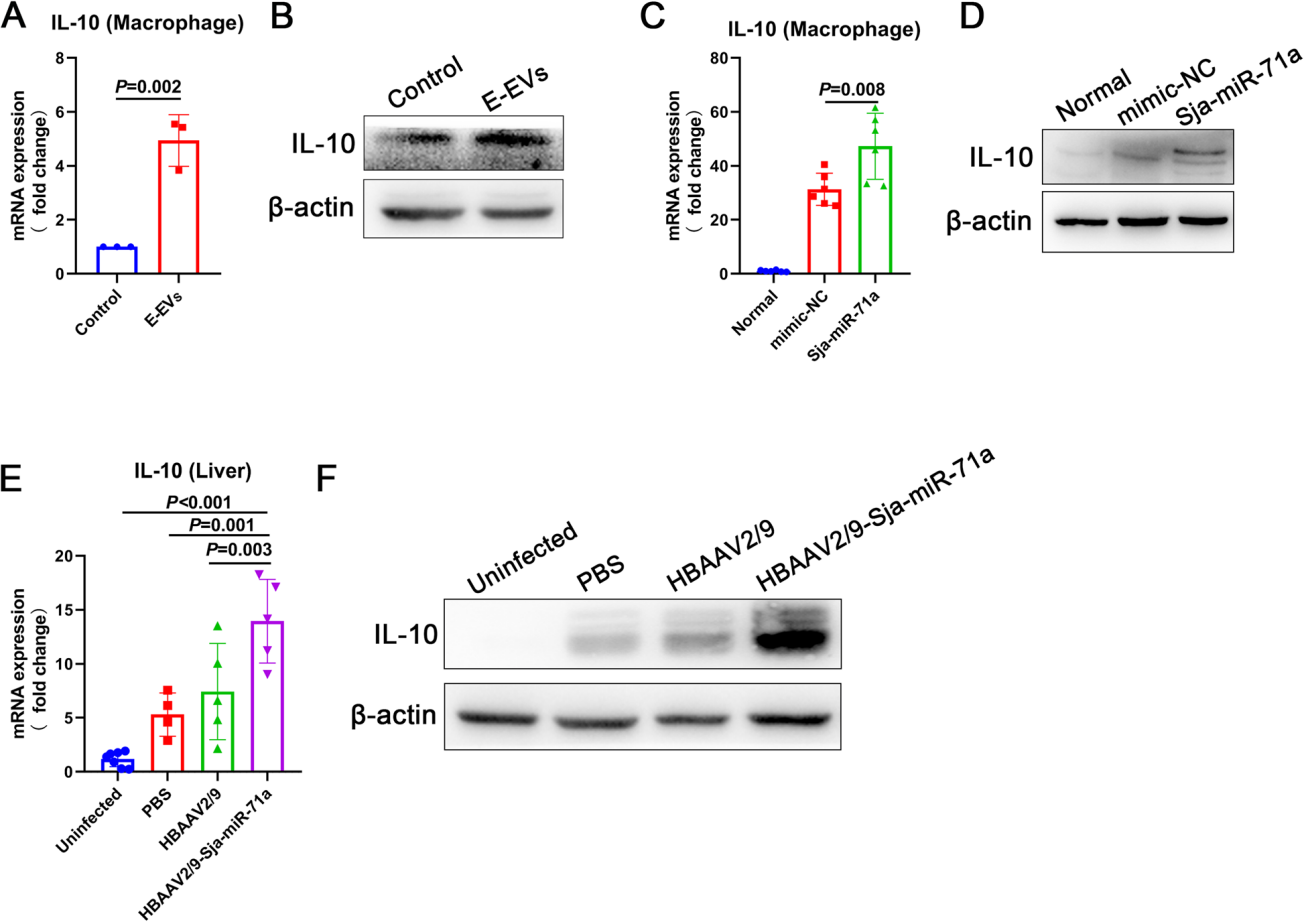
E-EVs and Sja-miR-71a up-regulate IL-10. **A** and **B** Macrophages were treated with E-EVs, and expression levels of IL-10 were analyzed with qRT-PCR and western blot. **C** and **D** Macrophages were treated with normal control (NC) mimic (50 nM, 24 h) and Sja-miR-71a (50 nM, 24 h). IL-10 expression was analyzed with qRT-PCR and western blot. **E** and **F** HBAAV2/9-Sja-miR-71a recombinant adeno-associated virus (rAAV) was constructed. Mice infected with *S. japonicum* were administered HBAAV2/9-Sja-miR-71a. Liver IL-10 expression was analyzed with qRT-PCR and western blot. A, representative of *n* = 3 independent experiments; C, representative of *n* = 6 independent experiments; E, *n* = 4-7 per group

Our previous study found that *S. japonicum* can up-regulate the expression of IL-10 in the host, which can inhibit the formation of NETs [16]. In order to explore the potential role of E-EVs and Sja-miR-71a in regulating IL-10 and subsequently inhibiting METs and NETs formation, we treated macrophages with E-EVs and Sja-miR-71a. We observed a significant up-regulation of IL-10 expression in macrophages following treatment with E-EVs (Fig. 3A, B). Similarly, treatment with Sja-miR-71a also led to an up-regulation of IL-10 expression in macrophages (Fig. 3C, D). In vivo experiments using HBAAV2/9-Sja-miR-71a further supported these findings, as IL-10 expression was significantly up-regulated in the livers of HBAAV2/9-Sja-miR-71a treated mice (Fig. 3E, F).

### Sja-miR-71a in E-EVs targets *Sema4D* to up-regulate IL-10, thereby inhibiting METs and NETs formation

**Fig. 4:**
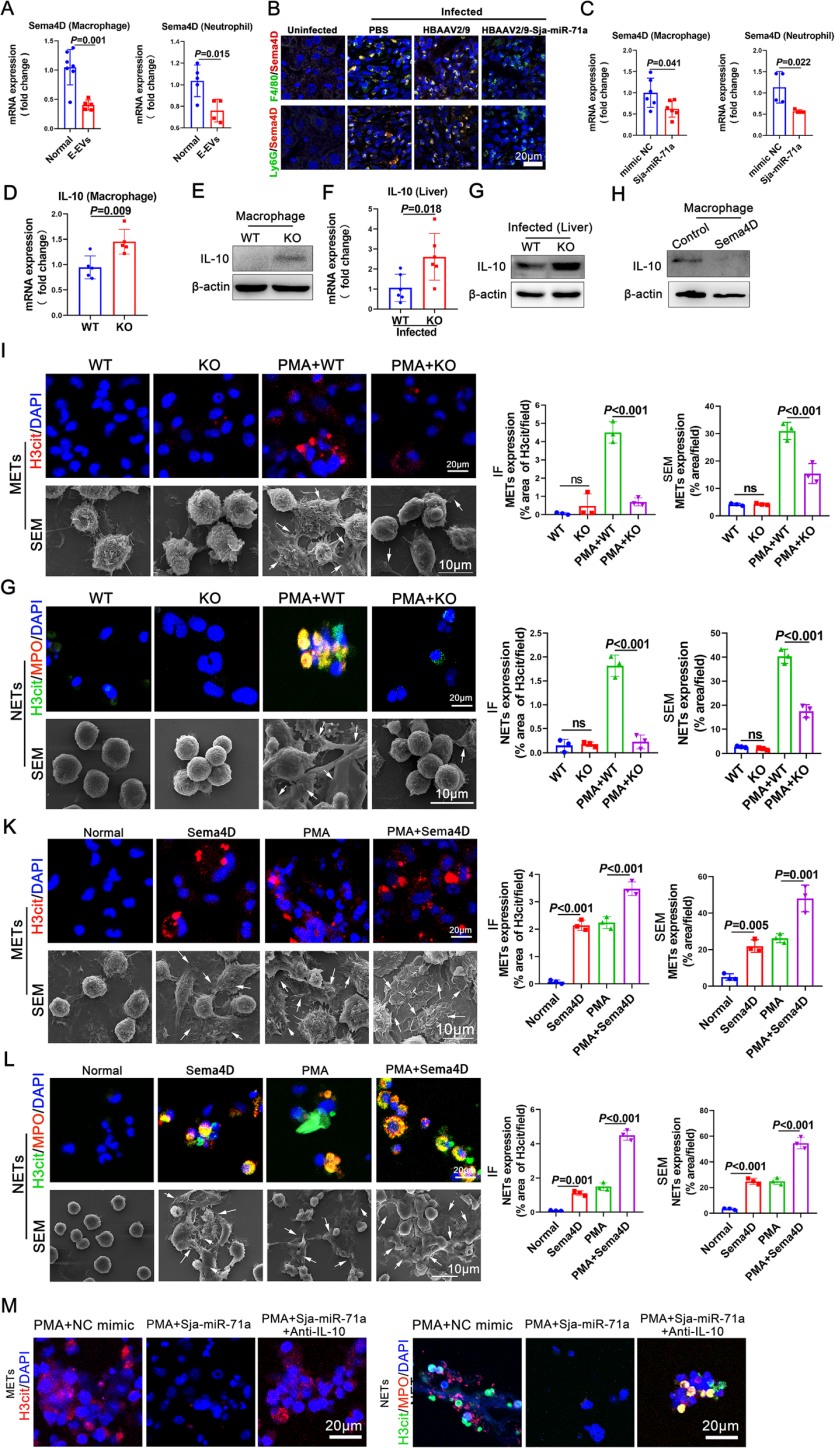
Sja-miR-71a in E-EVs targets *Sema4D* to up-regulate IL-10, thereby inhibiting METs and NETs formation. **A** Macrophages and neutrophils were isolated and treated with E-EVs, Sema4D expression was analyzed using qRT-PCR. **B** The expression of Sema4D in macrophages and neutrophils of the liver was detected by IF. **C** Macrophages and neutrophils were treated with Sja-miR-71a, Sema4D expression was analyzed using qRT-PCR. **D** and **E** IL-10 expression in *Sema4D* deletion (KO) and WT macrophages was analyzed using qRT-PCR and western blot. **F** and **G** IL-10 expression in the livers of *Sema4D* knockout (*Sema4D*-KO) and WT mice infected with *S. japonicum* were analyzed using qRT-PCR and western blot. **H** Macrophages were treated with recombinant Sema4D (10 μg/ml, 24 h), and the level of IL-10 was measured using western blot. **I** and **G** *Sema4D*-KO and WT macrophages and neutrophils were treated with PMA (500 nM, 5 h) or had no treatment at all. METs and NETs were observed using IF and SEM and quantified. **K** and **L** *Sema4D*-KO and WT macrophages and neutrophils were treated with recombinant Sema4D (10 μg/ml, 24 h), PMA (500 nM, 5 h), or PMA (500 nM, 5 h) + Sema4D (10 μg/ml, 24 h), METs and NETs were observed using IF and SEM and quantified. **M** Macrophages and neutrophils were treated with PMA (500 nM, 5 h) + NC mimic (50 nM, 24 h), PMA (500 nM, 5 h) + Sja-miR-71a (50 nM, 24 h), or PMA (500 nM, 5 h) + Sja-miR-71a (50 nM, 24 h) + IL-10 antibodies (15 μg/ml, 24 h), METs and NETs were observed using IF. Arrows: METs or NETs. **A** and **C,** *n* = 4-6 per group; **D**, *n* = 5 per group; **F**, *n* = 6 per group; **I-L** representative of *n* = 3 independent experiments

Sema4D, an important factor in *S. japonicum* infection, was directly regulated by Sja-miR-71a [13]. We isolated macrophages and neutrophils and then treated them with E-EVs. qRT-PCR revealed that E*-*EVs down-regulated the expression of Sema4D in macrophages and neutrophils (Fig. 4A). To explore the role of Sja-miR-71a in the downregulation of Sema4D expression in macrophages and neutrophils by E-EVs, we evaluated Sema4D in the livers of mice infected with *S. japonicum* and administered with HBAAV2/9-Sja-miR-71a. Notably, the levels of Sema4D in macrophages and neutrophils were decreased after treatment with HBAAV2/9-Sja-miR-71a (Fig. 4B). Furthermore, in vitro experiments found that the expression of Sema4D in macrophages and neutrophils was significantly down-regulated after treatment with Sja-miR-71a (Fig. 4C). We isolated macrophages from both wild-type (WT) and *Sema4D*^−/−^ mice and observed that the deletion of *Sema4D* led to an up-regulation of IL-10 expression in macrophages compared to the WT group (Fig. 4D, E). We then examined the effects of Sja-miR-71a targeting Sema4D on the inhibition of METs and NETs formation in *S. japonicum*-infected mice. Remarkably, the *Sema4D*-KO mice showed a significant increase in IL-10 expression in their livers (Fig. 4F, G). Furthermore, the treatment with recombinant Sema4D led to a down-regulation of IL-10 expression in macrophages (Fig. 4H). We also examined METs and NETs content in *Sema4D*-KO macrophages and observed a reduction in both PMA-induced METs formation (Fig. 4I) and PMA-induced NETs formation (Fig. 4G) compared to WT macrophages. Conversely, treatment with recombinant Sema4D resulted in a significant increase in METs and NETs formation and promoted PMA-induced METs and NETs formation (Fig. 4K, L). To further confirm that the down-regulation of METs and NETs by Sja-miR-71a is related to up-regulation of IL-10 by targeting *Sema4D*, we treated macrophages and neutrophils with IL-10 antibodies. We found that the inhibitory effect of Sja-miR-71a on PMA-induced METs and NETs formation was weakened after treatment with IL-10 antibodies (Fig. 4M). Collectively, these data suggest that Sja-miR-71a in E-EVs targets *Sema4D* to up-regulate IL-10, thereby inhibiting METs and NETs formation.

### Sja-miR-71a increases the expression of PPAR-γ

**Fig. 5:**
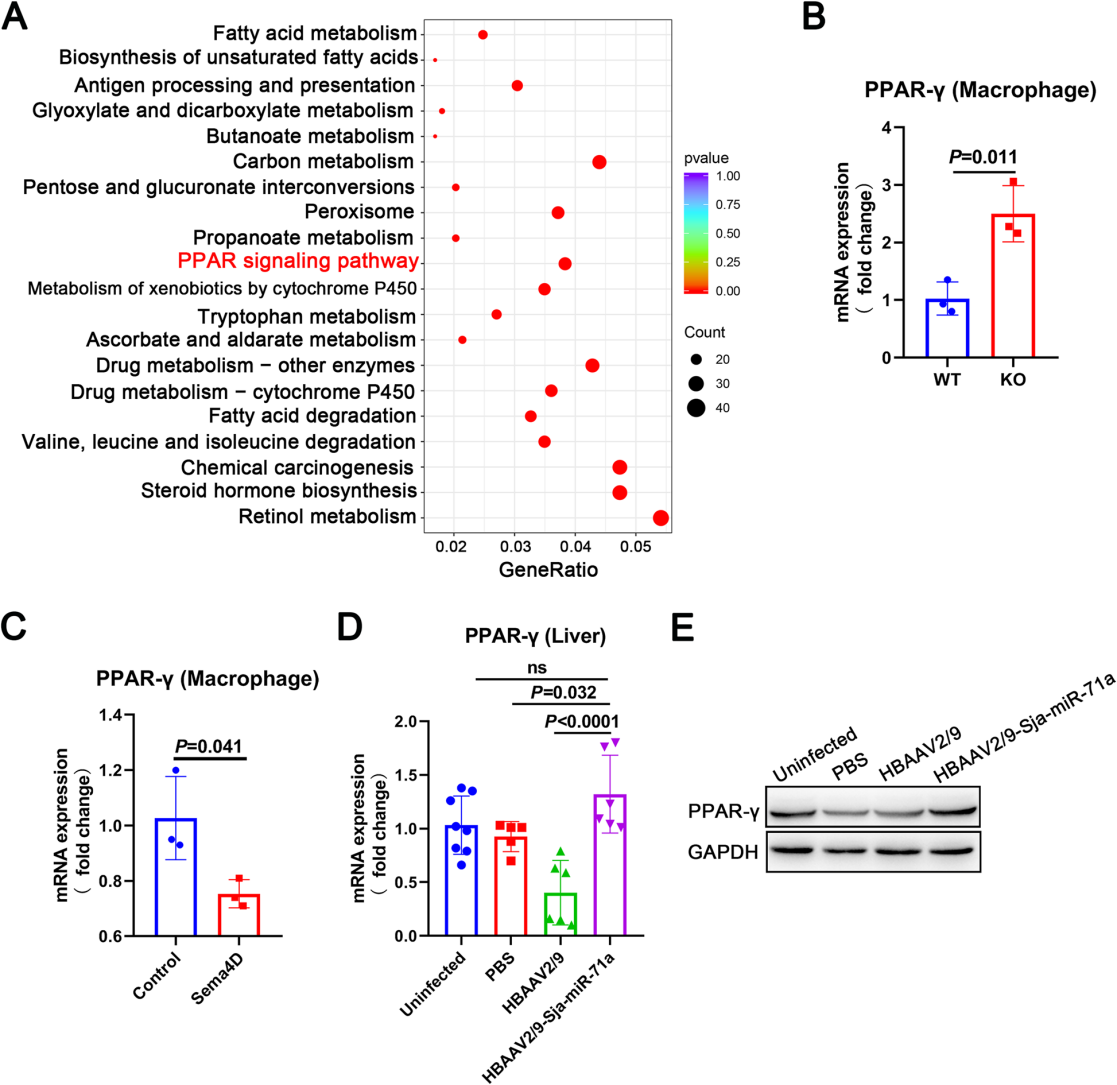
Sja-miR-71a increases the expression of PPAR-γ. **A** KEGG was used to analyze the up-regulated signaling pathway in *S. japonicum*-infected *Sema4D*-KO compared with *S. japonicum*-infected WT mice. **B** Expression levels of PPAR-γ in *Sema4D*-KO and WT macrophages were analyzed using qRT-PCR. **C** The PPAR-γ expression of macrophages treated with recombinant Sema4D (10 μg/ml, 24 h) was determined using qRT-PCR. **D** and **E** PPAR-γ expression in the livers of mice infected with *S. japonicum* was analyzed using qRT-PCR and western blot. **D**, *n* = 5-8 per group; **B**, **C**, representative of *n* = 3 independent experiments

To better understand if Sja-miR-71a in E-EVs targets *Sema4D* to up-regulate IL-10 and inhibit METs and NETs formation, we performed RNA sequencing and analysis of liver mRNAs obtained from *S. japonicum*-infected WT and *Sema4D*-KO mice. Comparative analysis of the global mRNA expression profiles revealed significant differences in the expression of 5072 genes (2253 up-regulated and 2819 down-regulated, *P* ≤ 0.05) between the *S. japonicum*-infected *Sema4D*-KO and WT mice (Fig. S4). The differential expression of genes (based on fold changes) between *S. japonicum*-infected *Sema4D*-KO mice and WT mice is represented as heatmaps in Fig. S5. Further analysis using the KEGG database identified the most significantly enriched pathways in *S. japonicum*-infected *Sema4D*-KO and WT mice, which included metabolic pathways, cytokine-cytokine receptor interactions, and the PPAR signaling pathway (Fig. S6). In particular, we discovered that the *S. japonicum*-infected Sema4D-KO mice exhibited up-regulated signaling pathways such as metabolic pathways and the PPAR signaling pathway, compared to the *S. japonicum*-infected WT mice (Fig. 5A). Previous studies have shown that PPAR-γ agonists are capable of upregulating IL-10 [20], and conversely, PPAR-γ antagonists or siRNA can effectively block the galangin-mediated upregulation of IL-10 [21].

Macrophages were then isolated from wild-type (WT) and *Sema4D* ^−/−^ mice and evaluated the expression of PPAR-γ. The deletion of *Sema4D* resulted in an up-regulation of PPAR-γ expression in macrophages compared to WT mice (Fig. 5B). Additionally, the expression of PPAR-γ in WT macrophages was reduced following treatment with recombinant Sema4D (Fig. 5C). We then analyzed the expression of PPAR-γ in the livers of infected mice treated with HBAAV2/9-Sja-miR-71a. The expression of PPAR-γ significantly increased after treatment with HBAAV2/9-Sja-miR-71a compared to the HBAAV2/9 group (Fig. 5D, E).

### Sja-miR-71a inhibits METs and NETs formation via the Sema4D/ PPAR-γ/ IL-10 axis

**Fig. 6:**
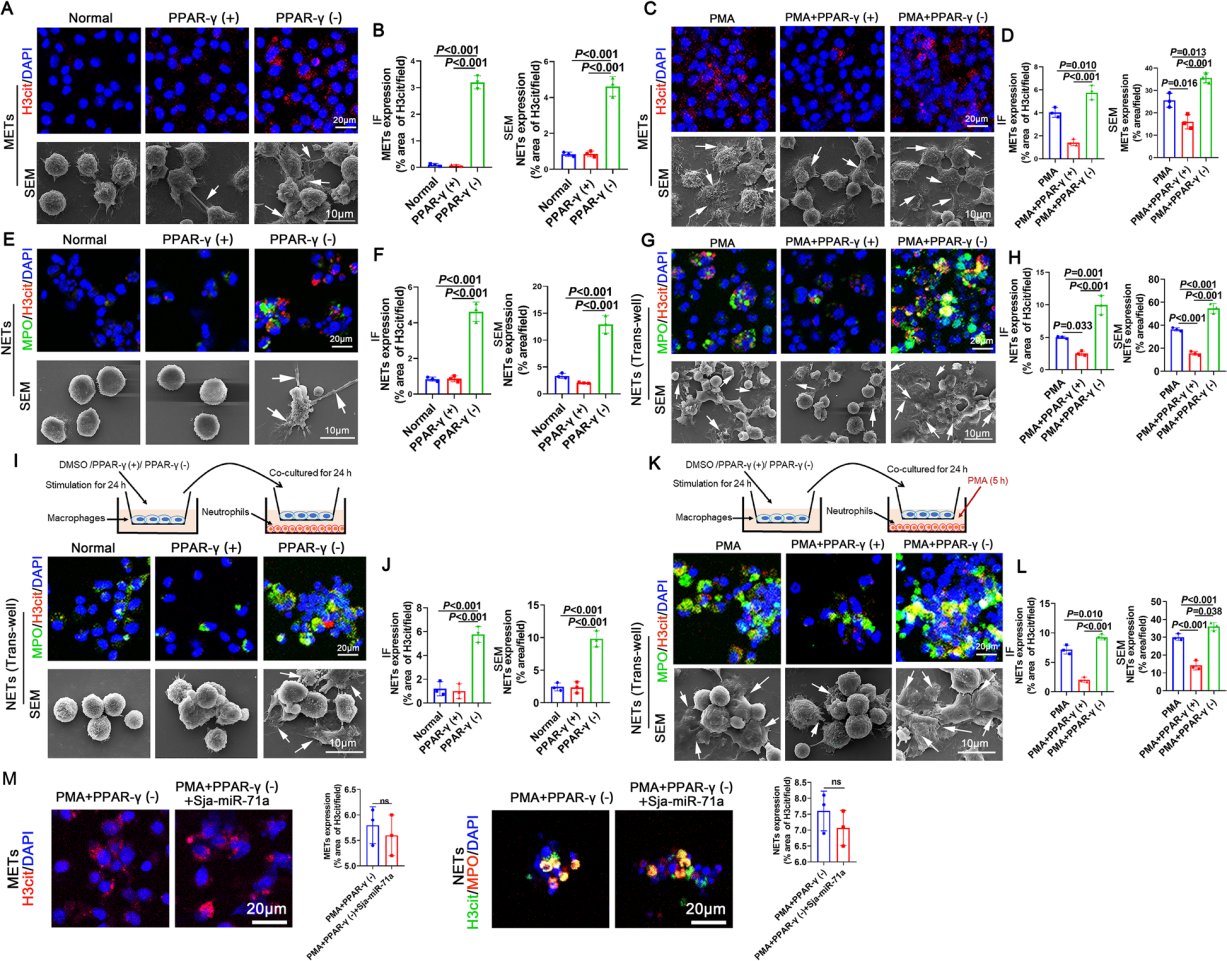
Sja-miR-71a inhibits METs and NETs formation via the *Sema4D*/ PPAR-γ/ IL-10 axis. **A-H** Macrophages and neutrophils were treated with a PPAR-γ agonist (30μΜ, 24 h), PPAR-γ antagonist (20μΜ, 24 h), PMA (500 nM, 5 h), PMA (500 nM, 5 h) + PPAR-γ agonist (30μΜ, 24 h), or a PMA (500 nM, 5 h) + PPAR-γ antagonist (20μΜ, 24 h). METs and NETs were observed (**A**, **C**, **E** and **G**), and quantified using IF and SEM (**B**, **D**, **F**, and **H**). **I** and **J** Macrophages and neutrophils were treated with a PPAR-γ agonist (30μΜ, 24 h) AND PPAR-γ antagonist (20μΜ, 24 h) (DMSO as control), then co-cultured for 24 h via trans-well, NETs were observed using IF and SEM (**I**) and quantified (**J**). **K** and **L** Macrophages and neutrophils were treated with a PPAR-γ agonist (30μΜ, 24 h) AND PPAR-γ antagonist (20μΜ, 24 h) (DMSO as control), then co-cultured for 24 h via trans-well. Neutrophils were treated with PMA (500 nM) for the last 5 hours, NETs were observed using IF and SEM (**K**) and quantified (**L**). **M** Macrophages and neutrophils were treated with PMA, PPAR-γ antagonist, and Sja-miR-71a simultaneously, METs and NETs were observed using IF. Arrows: METs or NETs. Representative of *n* = 3 independent experiments

We then explored whether activating PPAR could upregulate IL-10 expression, thus leading to the suppression of METs and NETs formation. We observed that MET formation was increased following treatment with a PPAR-γ antagonist (Fig. 6A, B). A PPAR-γ agonist inhibited PMA-induced METs formation, while a PPAR-γ antagonist promoted PMA-induced METs formation (Fig. 6C, D). Similarly, the formation of NETs was enhanced in the presence of a PPAR-γ antagonist (Fig. 6E, F), while a PPAR-γ agonist inhibited PMA-induced NETs formation, and a PPAR-γ antagonist promoted PMA-induced NETs formation (Fig. 6G, H). Furthermore, to further elucidate the role of PPAR-γ in METs and NETs formation, we activated or inhibited PPAR-γ in macrophages using agonists and antagonists, followed by co-culturing these macrophages with neutrophils using a trans-well system. Interesting observations revealed that co-culturing with PPAR-γ-inhibited macrophages led to an increase in NETs formation (Fig. 6I, J), and PPAR-γ inhibited macrophages promoted PMA-induced NETs formation (Fig. 6 K, L). Conversely, PPAR-γ activated macrophages inhibited PMA-induced NETs formation (Fig. 6 K, L). To further confirm that the down-regulation of METs and NETs by Sja-miR-71a is related to the activation of PPAR-γ, we treated macrophages and neutrophils with PMA, PPAR-γ antagonist, and Sja-miR-71a simultaneously. We found that the inhibitory effect of Sja-miR-71a on PMA-induced METs and NETs formation was weakened after treatment with PPAR-γ antagonist (Fig. 6 M). Taken together, these results suggest that Sja-miR-71a inhibits METs and NETs formation via the *Sema4D*/ PPAR-γ/ IL-10 axis.

## Discussion

In this study, we demonstrate that *S. japonicum* eggs inhibit the formation of METs and NETs through the secretion of EVs. We also identified the involvement of Sja-miR-71a in the E-EV-mediated inhibition of METs and NETs. Furthermore, we elucidated that Sja-miR-71a in E-EVs inhibits METs and NETs formation via the *Sema4D*/ PPAR-γ/ IL-10 axis.

The immune defense mechanisms mediated by METs and NETs play a crucial role in host defense against various pathogens. Previous studies have shown that both METs and NETs possess microbicidal activities against bacteria, fungi, and viruses. METs, for example, have been found to exhibit potential microbicidal activity against pathogens such as *Staphylococcus aureus*, *Streptococcus agalactiae*, *Escherichia coli,* and *Candida albicans* [22–25]. NETs can trap, neutralize, and kill bacteria [26], fungi [27], and viruses [9], and are thought to prevent bacterial and fungal dissemination [28, 29]. Additionally, NETs have been reported to inhibit parasites as well [17, 30]. Previous work by our group found that host liver-derived extracellular vesicles deliver miR-142a-3p, which induces NETs to block the development of *S. japonicum* by targeting WASL [31]. While the host uses ETs to kill pathogens, bacteria, protozoa, and fungi have all reportedly evolved strategies to overcome NETs trapping by secreting nucleases to degrade the DNA backbone of these structures [32–35]. Hookworm larvae are able to mitigate the effect of NETs by secreting a deoxyribonuclease (NbDNase II) to degrade its DNA backbone [17]. Similarly, in this study, we found that *S. japonicum* eggs inhibit the formation of METs and NETs through the secretion of EVs.

EVs are a heterogeneous group of membranous structures that facilitate the transport of bioactive molecules [36]. The membrane structure of EVs allows for the long-distance transportation of such molecules without rapid degradation. Compared to direct transport, EV-mediated transport of parasite molecules is more efficient. In our study, we found that EVs derived from *S. japonicum* eggs (E-EVs) actively inhibit the formation of METs and NETs. This observation helps explains why the host is only able to block the development of *S. japonicum* through these defense mechanisms but cannot completely eliminate the parasite. As a result, *S. japonicum* eggs can persist within the host for an extended period and successfully evade attacks from immune cells before being released from the host’s body.

MiR-71 is a bilaterian miRNA that plays an important role in helminths but is absent in vertebrate hosts [37]. In *Caenorhabditis elegans*, miR-71 mediates the age-dependent opposing contributions of the stress-activated kinase KGB-1, and miR-71 inhibits calcium signaling by targeting the TIR-1/Sarm1 adaptor protein to control stochastic L/R neuronal asymmetry [38, 39]. MiR-71 plays an important role in *Echinococcus* development [37]. In the *S. japonicum*, miR-71 is related to the sexual development of the parasite [40]. Our previous study demonstrated that E-EVs suppress liver fibrosis by delivering Sja-miR-71a [13]. In the current study, we found that Sja-miR-71a carried by EVs plays a key role in the E-EV-mediated inhibition of METs and NETs. This further demonstrated the importance of miR-71 in the survival of helminths.

The immune response involving METs and NETs can be influenced by various factors. Saitoh et al. showed that HIV-1 can be captured by NETs and subsequently eliminated through myeloperoxidase and a-defensin. However, HIV-1 counteracts this response by inducing the production of interleukin IL-10, which inhibits NETs formation [9]. In our previous study, we discovered that *S. japonicum* up-regulates the expression of host IL-10, which also inhibit the formation of NETs [16]. In this study, we found that E-EVs and Sja-miR-71a carried by E-EVs upregulate the expression of IL-10, suggesting that the inhibition of NETs formation by E-EVs and Sja-miR-71a is related to the upregulation of IL-10.

*Sema4D* is a direct target of Sja-miR-71a [13]. We found that *Sema4D* deletion upregulates the expression of IL-10 and inhibits the formation METs and NETs. Moreover, PPAR-γ agonists have been shown to upregulate IL-10 expression [20], while PPAR-γ antagonists or siRNA significantly inhibit the galangin-mediated upregulation of IL-10 [21]. Interestingly, we found that Sja-miR-71a upregulates PPAR-γ, and the level of PPAR-γ in *Sema4D*-KO mice was significantly higher than in WT mice. Additionally, PPAR-γ activation decreased the formation of METs and NETs induced by PMA, and PPAR-γ activated macrophages can further inhibit NETs formation. Based on these findings, we conclude that Sja-miR-71a in E-EVs inhibits the formation of METs and NETs through the Sema4D/PPAR-γ/IL-10 axis. Schistosome eggs can potentially facilitate immune evasion by inhibiting the formation of METs and NETs. However, further research is required to investigate the impact of METs and NETs on the survival of Schistosomes within the host and their ability to produce eggs.

## Conclusion

In summary, our study provides evidence that Sja-miR-71a delivered by E-EVs inhibits host METs and NETs formation through the targeting of *Sema4D*. We further demonstrated that Sja-miR-71a inhibits METs and NETs formation via the *Sema4D*/PPAR-γ/IL-10 axis. These results contribute to further understanding the molecular mechanisms underlying *Schistosoma*–host interactions.

### Supplementary Information

**Additional file 1.** Figures S1-S6 and Table S1.

**Additional file 2.** Original western blots.

## Data Availability

The data presented in this study are available on request from the corresponding author.
