# Supplementary material for: Schistosome egg-derived extracellular vesicles deliver Sja-miR-71a inhibits host macrophage and neutrophil extracellular traps via targeting Sema4D
Source: Cell Commun Signal. 2023 Dec 21;21:366. doi: 10.1186/s12964-023-01395-8 (PMC10734185; doi:10.1186/s12964-023-01395-8)
Supplement: Supplementary file 2 — Additional file 1. Figures S1-S6 and Table S1. [file 12964_2023_1395_MOESM1_ESM.docx]

**SUPPLEMENTARY DATA**

**Supplementary Figures**

**
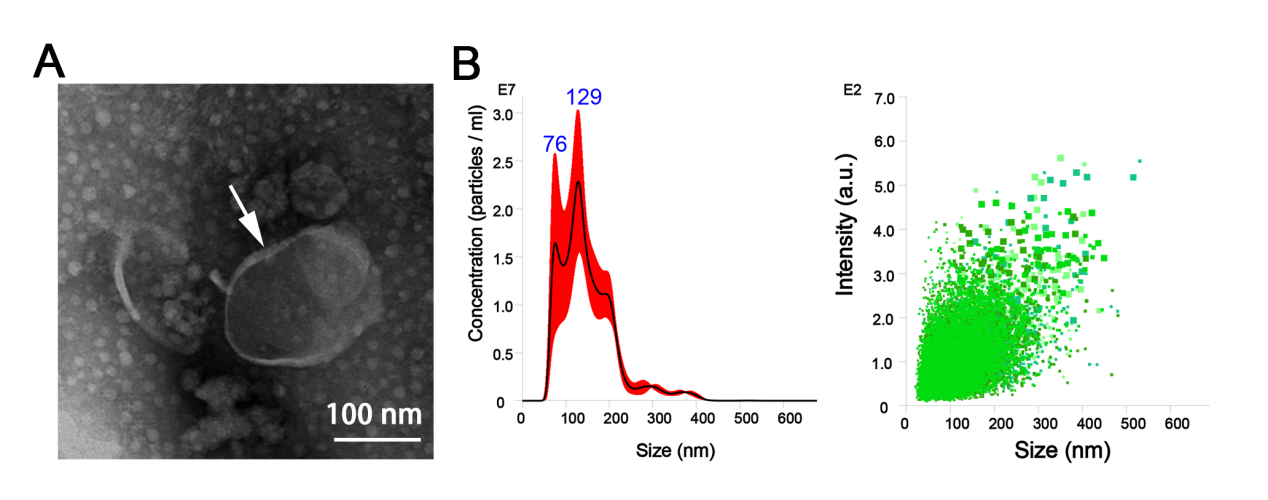
**

**Fig. S1 *S. japonicum* egg-derived extracellular vesicles (E-EVs) were isolated from the culture supernatant of *S. japonicum* eggs and analyzed using negative-staining transmission electron microscopy and nanoparticle tracking analysis. (A)** E-EVs were purified and analyzed using negative-staining transmission electron microscopy. (Arrow: E-EVs). (**B**) E-EVs particles were studied using nanoparticle tracking analysis .

**
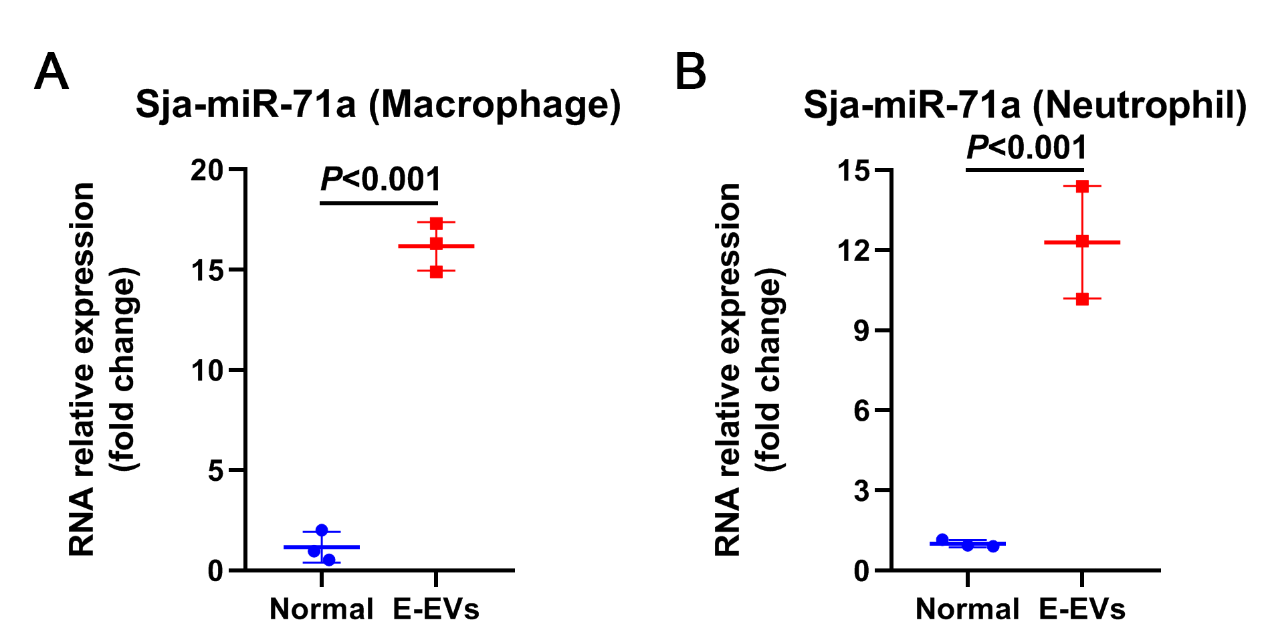
**

**Fig. S2 E-EVs deliver Sja-miR-71a to macrophages and neutrophils.** Macrophages (**A**) and neutrophils (**B**) were treated with E-EVs (10 μg/mL, 24 h), Sja-miR-71a in macrophages and neutrophils was analyzed with qRT-PCR.


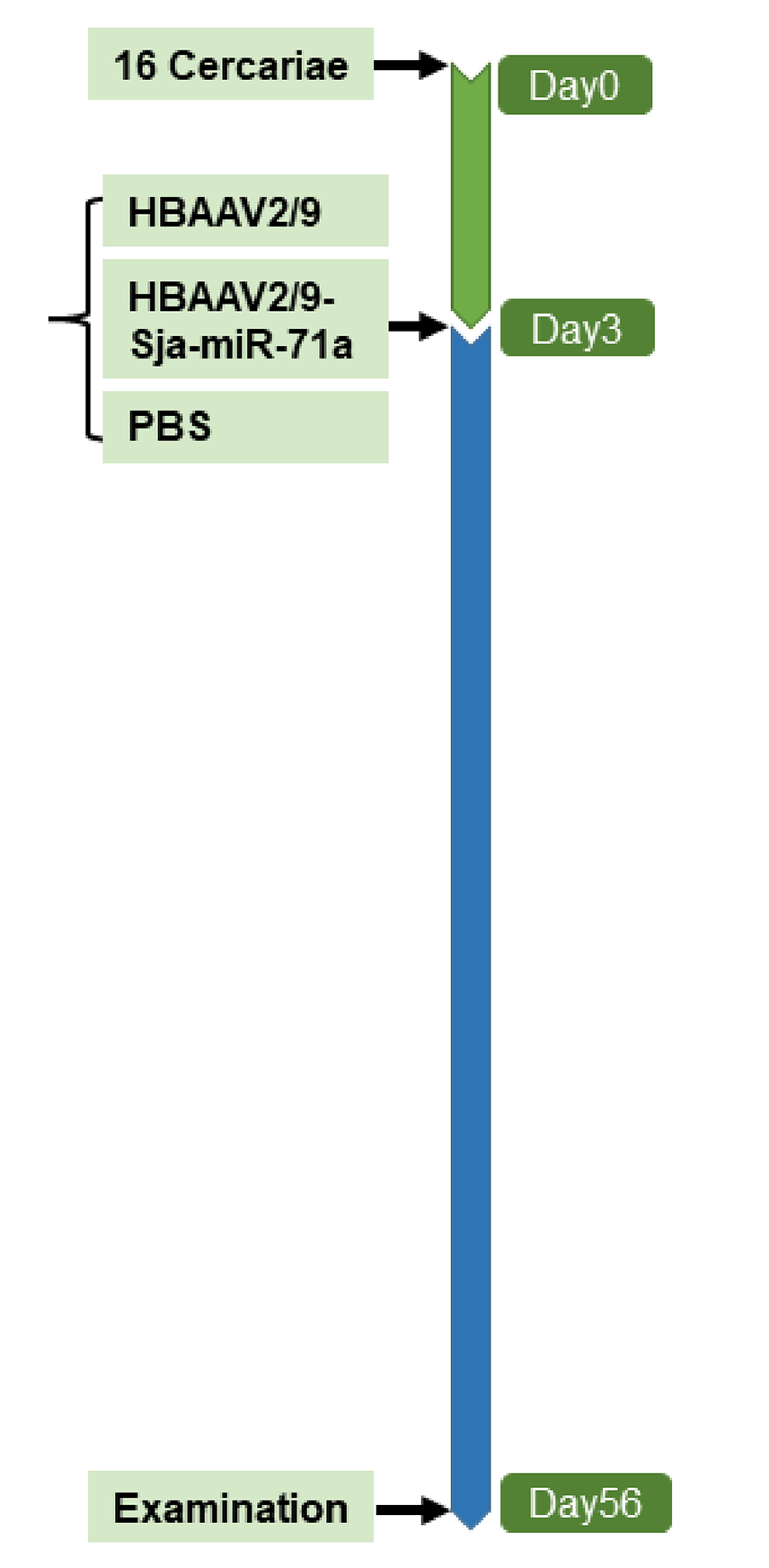


**Fig. S3 Timing of parasite infection, intravenous injection of rAAV vectors or PBS, and sample examination.**


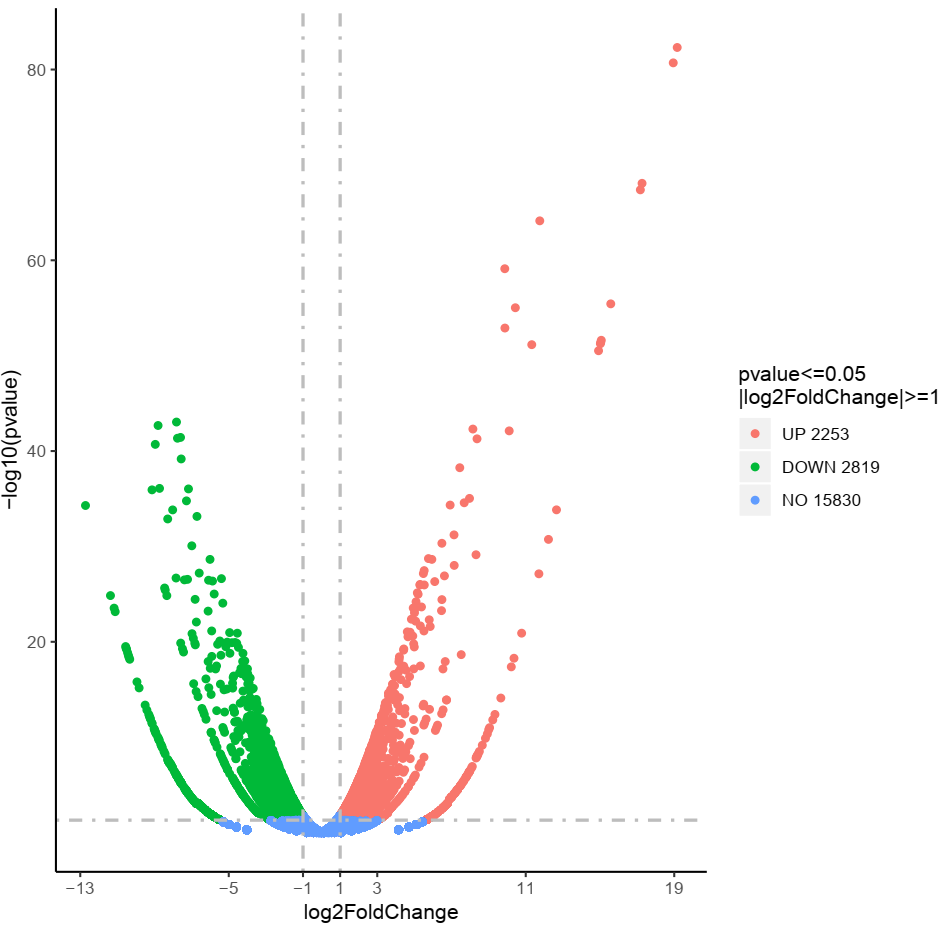


**Fig. S4** Comparative assessment of the global mRNA expression profiles of various genes in *S. japonicum*-infected Sema4D -KO and WT mice identified 5072 genes (2253 up-regulated and 2819 down-regulated, *P*≤0.05) that had significantly different expression levels between groups.


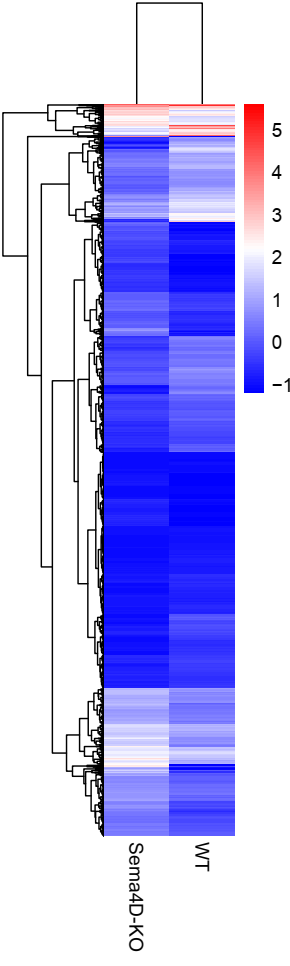


**Fig. S5** Genes that expressed differently (based on fold changes) between *S. japonicum*-infected Sema4D -KO and WT mice shown on a heatmap.


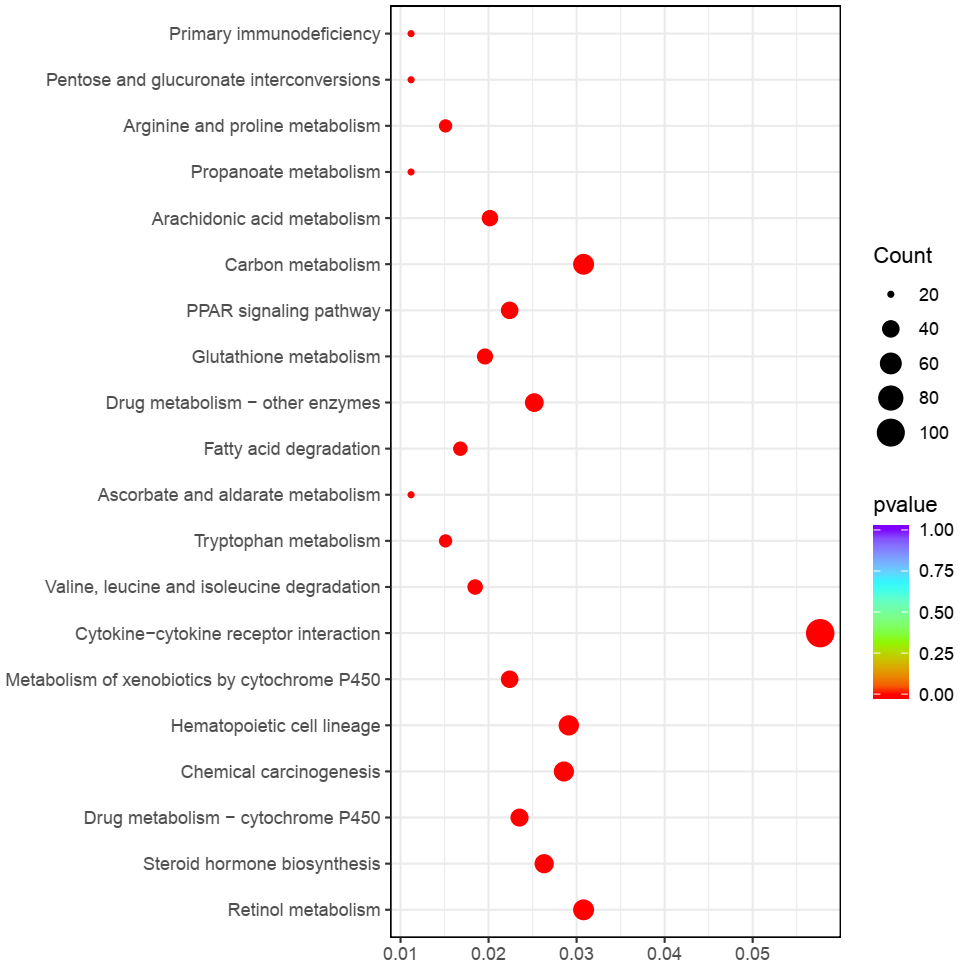


**Fig. S6** KEGG was used to identify the biological pathways that were most significantly enriched in *S. japonicum*-infected Sema4D -KO and WT mice. The 20 most significantly enriched pathways included metabolic pathways, cytokine−cytokine receptor interactions, and the PPAR signaling pathway.

**Supplementary Table**

**Table S1 Quantitative Real Time PCR Primer Sequences**

| Gene | Forward (5’-3’) | Reverse (5’-3’) |
| --- | --- | --- |
| IL-10 | AGTACAGCCGGGAAGACAAT | TCTAGGAGCATGTGGCTCTG |
| PPAR-γ | CTCCAAGAATACCAAAGTGCGA | GCCTGATGCTTTATCCCCACA |
| Sema4D | CTGCTACAAGGGCTACCTGC | GCTCGACCAGTGTCTCCTTC |
| GAPH | ACTCCACTCACGGCAAATTC | TCTCCATGGTGGTGAAGACA |
