# Supplementary figures and images for: Schistosome egg-derived extracellular vesicles deliver Sja-miR-71a inhibits host macrophage and neutrophil extracellular traps via targeting Sema4D
Source: Cell Commun Signal. 2023 Dec 21;21:366. doi: 10.1186/s12964-023-01395-8 (PMC10734185; doi:10.1186/s12964-023-01395-8)

**Original western blots**

Fig. 3B


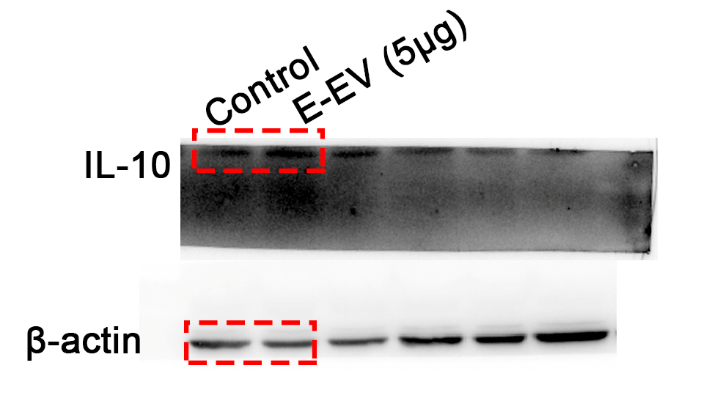


Fig. 3D


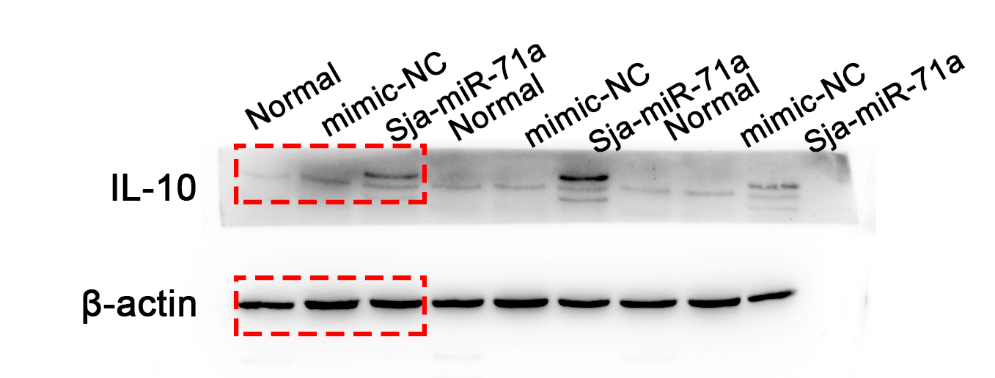


Fig. 3F


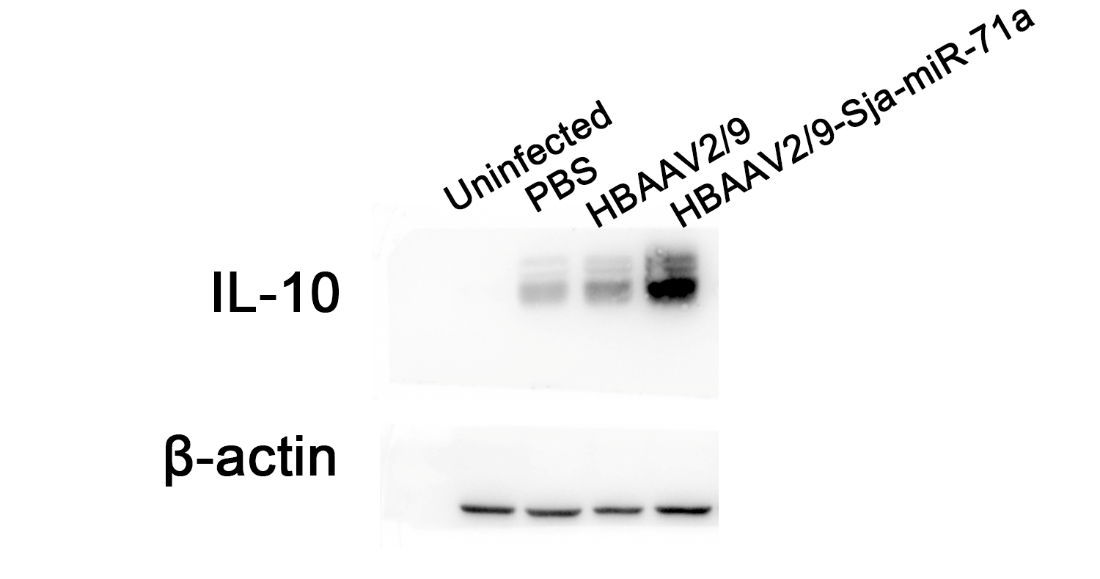


Fig. 4B


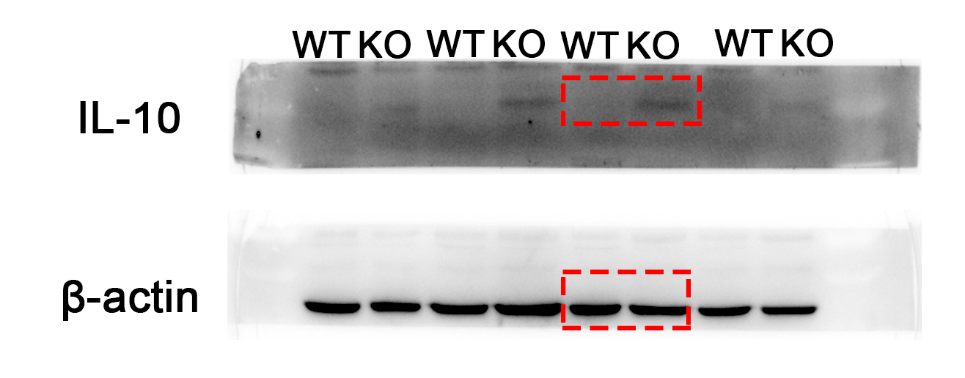


Fig. 4D


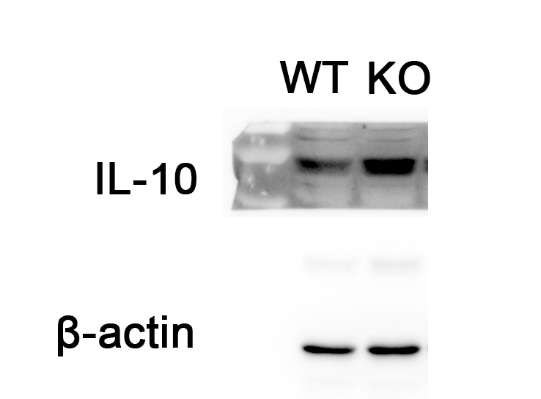


Fig. 4E


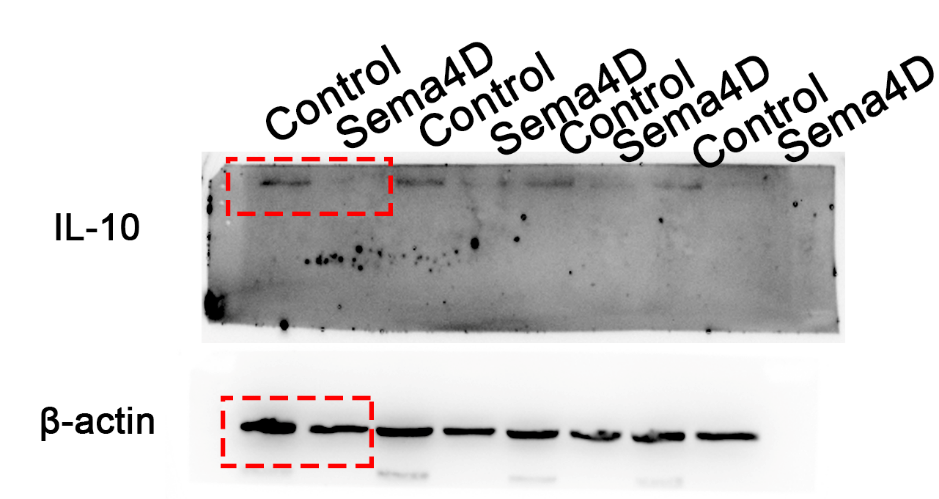


Fig. 5E


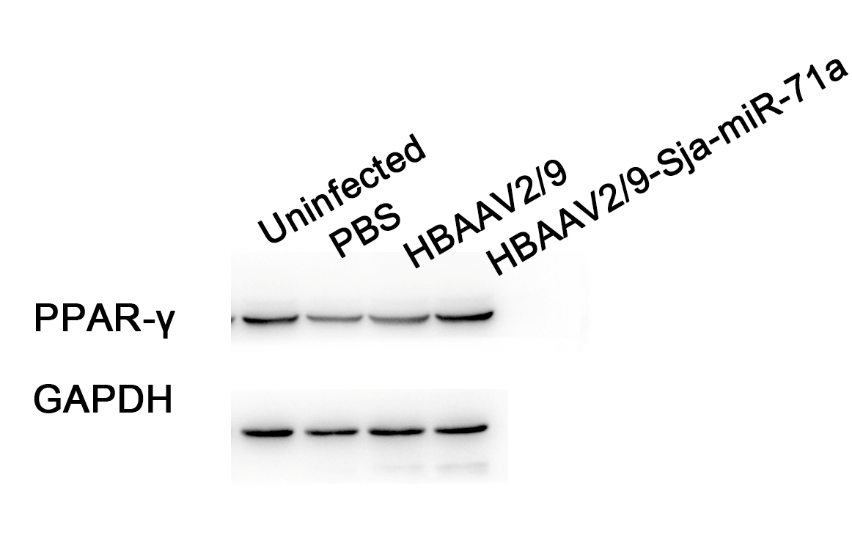

Supplement: Supplementary file 3 — Additional file 2. Original western blots. [file 12964_2023_1395_MOESM2_ESM.docx]
